# Comparison of strategies for daily surveillance of international travellers quarantined in Vanuatu, October–December 2020

**DOI:** 10.5365/wpsar.2022.13.2.918

**Published:** 2022-06-30

**Authors:** Caroline van Gemert, Wendy Williams, Joanne Mariasua, Debbie Fred, Matthew Cornish, Len Tarivonda, Posikai Samuel Tapo, Vincent Atua, Obed Manwo, Philippe Guyant, Lola Iavro, Geoff Clark

**Affiliations:** a Vanuatu Health Program, Port Vila, Vanuatu.; b Melbourne School of Population and Global Health, The University of Melbourne, Melbourne, Victoria, Australia.; c Burnet Institute, Melbourne, Victoria, Australia.; d Vanuatu Ministry of Health, Port Vila, Vanuatu.; e Private physician, Port Vila, Vanuatu.; f Vila Central Hospital, Port Vila, Vanuatu.; g Shefa Provincial Health Service, Port Vila, Vanuatu.; h World Health Organization Country Liaison Office in Vanuatu, Port Vila, Vanuatu.

## Abstract

**Objective:**

To prevent importation of coronavirus disease 2019 (COVID-19) to Vanuatu, since March 2020, all travellers to the country have been required to complete a 14-day quarantine in a government-designated facility. A short message service (SMS, or “text message”) system was developed to collect information on symptoms of COVID-19 among travellers in quarantine. A trial within a cohort study was conducted among travellers arriving to Vanuatu by air from 27 October to 7 December 2020 to assess SMS acceptability, efficiency and utility and whether SMS-based health monitoring was as effective as in-person monitoring in identifying people with COVID-19 symptoms.

**Methods:**

Control group participants received standard monitoring (daily in-person visits) and participants in the intervention group received a daily SMS text requesting a response coded for symptom development. Differences between the two groups were determined using χ^2^ tests.

**Results:**

Of the 495 eligible travellers, 423 participated; 170 were allocated to the control group and 253 to the intervention group. At least one return SMS text was received from 50% (107/212) of participants who were confirmed to have received an SMS text. Less than 2% (4/253) of the intervention group and 0% of the control group reported symptoms.

**Discussion:**

The SMS intervention had a high level of acceptability. SMS is a useful tool to monitor symptom development among people in quarantine and for broader public health programmes that require follow up.

Since March 2020, the Government of Vanuatu (a group of 83 islands with a population of approximately 302 000) has implemented several measures to prevent the importation and community transmission of severe acute respiratory syndrome coronavirus 2 (SARS-CoV-2), the pathogen that causes coronavirus disease 2019 (COVID-19). Measures include quarantining incoming travellers and suspending international ports of entry into Vanuatu. (*1*) Between May and December 2020, 4661 people returned to Vanuatu through a repatriation programme. Implementing quarantine and monitoring the symptoms of those in quarantine added a considerable burden to the public health system in Shefa Province where the capital city, Port Vila, is located. Public health and nursing staff were redeployed from community-based health services within Shefa and from other provincial health services, resulting in the closure of health centres or delays in providing some essential health services. The impact of these closures and delays has not been quantified.

To address the issue of limited health resources and high demand on public health resources due to these quarantine requirements, Shefa Provincial Health Service and the Vanuatu Ministry of Health (MoH) considered alternative strategies for monitoring the health of asymptomatic people in quarantine, such as using short message service (SMS, or “text message”) technology. SMS technology has been effectively used in a variety of public health programmes aiming to control infectious diseases; for example, SMS has been used to send reminders for infant vaccinations, (*2*, *3*) to send treatment reminders to improve adherence to HIV pre-exposure prophylaxis (*4*) and tuberculosis treatment, (*5*) and to send partner notifications for contact tracing for sexually transmitted infections. (*6*) Limited studies have used SMS texts to record two-way information flow (that is, responses to questions that are recorded within the SMS), and none of these has been from a Pacific island country. For example, an intervention in Western Australia used SMS to monitor the health of people who had recently travelled to areas in Africa affected by Ebola virus disease. (*7*) In that intervention, people were sent an SMS text twice daily for 3 weeks requesting their temperature and information about symptoms. If any related symptoms were reported, a medical officer contacted the person and followed up as appropriate.

The present study sought to develop and assess an SMS-based system to streamline active monitoring of travellers returning to Vanuatu. The objective of the study was to assess SMS acceptability, efficiency and utility and whether SMS-based health monitoring was as effective as in-person monitoring to identify people with symptoms of COVID-19 in Vanuatu.

## Methods

### Study design

This operational research used a trial within a cohort. The cohort was inbound travellers to Vanuatu who arrived by air between 27 October and 7 December 2020.

### Inclusion and exclusion criteria

Individuals aged (*3*)18 years arriving from a country classified as low risk were eligible to participate. Low-risk countries were defined by the Vanuatu MoH and included those with a 14-day incidence of < 10 newly reported cases/100 000 population that had direct flights to Vanuatu and had no reported community transmission. Individuals with pre-existing respiratory conditions or health issues were identified through review of pre-travel registration forms, as per standard processes, and were excluded from the study.

### Recruitment and consent

A research nurse obtained written consent from participants after they arrived at their hotel; consent forms were translated into both English and Bislama (one of the official languages of Vanuatu).

### Infection prevention and control measures

The research nurse wore personal protective equipment based on World Health Organization (WHO) recommendations for health-care workers. (*8*) Where possible, the research nurse contacted eligible participants by phone. Consent forms were collected by the research nurse and stored in a file for 3 days before being accessed.

### Control and intervention groups

In the control group, participants received standard monitoring. This involved daily visits from public health teams. Monitoring teams worked in pairs, with one member recording data and the other conducting the assessment. The assessment included a temperature check using a digital thermometer and enquiries about symptoms such as fever, cough, sore throat or related symptoms. The results were recorded in a paper record that was stored at Shefa Provincial Health Service before the data were entered into the quarantine database (a customized Google Sheets database with restricted access).

Participants with symptoms were followed up by a rapid response team. If asymptomatic, people in quarantine were tested on days 5 and 11 after arrival, and if symptomatic, they were tested on the day of symptom onset. Laboratory testing was conducted using the Xpert Xpress SARS-CoV-2 cartridge assay (Cepheid, Sunnyvale, CA, USA). Data on medical follow up and outcomes (including testing and associated results) were not included in the quarantine database and were stored in the District Health Information Software Version 2 (DHIS2) COVID-19 case-based surveillance module.

Participants in the intervention group were monitored only by SMS. Participants without a local mobile telephone number were provided with a local SIM card and were requested to activate the card. During the quarantine period, each day at approximately 09:00, an SMS text was sent to the person in quarantine: “In the last 24 hours, have you experienced a fever, cough or shortness of breath? If no, reply 1. If yes, reply 2.”

The returned SMS data were stored in a cloud-based database managed by Digicel Vanuatu (a mobile phone service provider) and accessible to the MoH. Data were downloaded as Microsoft Excel files and linked to the research database monitored by the Ministry. If symptoms were reported, the database automatically generated an alert that was sent to a public health officer. The officer then telephoned the individual to verify symptoms and confirm their location. If the symptoms met the COVID-19 case definition (based on the WHO case definition), (*9*) the Shefa provincial public health manager was informed, and the provincial rapid response team, comprising a medical practitioner and public health officer, was activated. Data on medical follow up and outcomes (including testing and associated results) were not included in the quarantine database and were stored in the DHIS2 COVID-19 case-based surveillance module. Participants in the intervention group were also tested on days 5 and 11 after arrival, as per the processes described above.

### Sample size and allocation

The sample size required to have sufficient power to detect 5% of the population reporting fever, cough or shortness of breath with a design effect of 2 was 540. All arrivals reporting travel from an eligible location were recruited until the sample size was reached. Participants were block-allocated to either the control or intervention group according to date of arrival and hotel of allocation. Block randomization was used to ensure that all participants in the control group were quarantined in the same hotel, therefore reducing strain on the public health workforce by reducing their need to travel between multiple hotels.

### Data collected

Relevant data were extracted from the quarantine database for participants in the control and intervention groups, including name, date of birth, port of departure and date of arrival in Vanuatu, and these were transferred to the research database (a customized Microsoft Excel database) and stored in a secure folder. The research database included additional data about participants in the intervention group including whether an outbound SMS text was sent by the Vanuatu MoH (yes/no) and the date, whether an inbound SMS text was received by the Vanuatu MoH (yes/no), the contents of the SMS response (1 = no symptoms, 2 = symptoms), whether a SARS-CoV-2 test was requested (yes/no) and the results of the test. In addition, on the final day before release from quarantine, a five-question paper-based survey was distributed to all participants in the intervention group to assess the acceptability of the SMS monitoring system.

### Data analysis

χ^2^ tests were used to compare: (1) characteristics of participants versus non-participants, (2) characteristics of the intervention group versus control group, (3) response rates of the intervention group by demographic variables and (4) symptoms reported in the intervention group versus control group. A *P*-value of < 0.05 was considered statistically significant. Stata Standard Edition software (version 15; College Station, TX, USA) was used for statistical analyses.

## Results

### Participation rates

The last inbound flight to Vanuatu for 2020 occurred on 7 December 2020, and therefore the target sample size of 540 participants could not be reached. However, among 495 people eligible to participate, 423 consented (participation rate of 85%).

There was no statistical difference in proportions among participants and non-participants by sex and nationality, but more participants were aged 18–40 years compared with non-participants (*P* = 0.002). These proportions reflect the people travelling to Vanuatu at that time. Three quarters (78.0%; 330/423) of participants were male, one quarter (26.2%; 111/423) was aged (*3*)40 years and nearly all (95.0%; 402/423) were Vanuatu nationals. The nationalities of the 21 people who were not Vanuatu citizens included Australian (*n* = 13), French (*n* = 6), Chinese (*n* = 1) and Fijian (*n* = 1).

### Description of intervention and control groups

Among the 423 participants, 170 (40.2%) were allocated to the control group and 253 (59.8%) to the intervention group. The control and intervention groups had similar distributions among age group and nationality; however, fewer males were in the control group (71.8% [122] versus 82.2% [208], *P* = 0.011) (**Table 1**).

**Table 1:** Characteristics of participants

Characteristic	No. (%) of participants		*P* ^a^
	Control group	Intervention group	
Total	170 (40.2)	253 (59.8)	-
Sex			
Female	48 (28.2)	45 (17.8)	0.011
Male	122 (71.8)	208 (82.2)	
Age group (years)			
18–40	119 (70.0)	193 (76.3)	> 0.05
> 40	51 (30.0)	60 (23.7)	
Nationality			
Vanuatu	159 (93.5)	243 (96.0)	> 0.05
Other	11 (6.5)	10 (4.0)	

^a^ χ^2^ tests were used to compare the characteristics of the intervention group with those of the control group. *P* < 0.05 was considered statistically significant.

### Intervention response rate

Among the 253 participants in the intervention group, evidence that an SMS was received by the participant was available for 212 participants (83.8%). The reason why an SMS text was not received is unclear, but is likely due to non-registration of the SIM card or another technical issue. Among these 212 participants, at least one return SMS text was received from a total of 107 participants (50.4%). There was no statistical difference (*P* > 0.05) in sex, age group or nationality for participants in terms of the number of responses received (**Table 2**). The response rate varied for the first 3 days by sex, but ranged between 35% and 50% for all participants each day between day 4 and day 14 (data not shown).

**Table 2:** Characteristics of the 212/253 people in the intervention group for whom there was evidence that an SMS (short message service) text was received requesting information about their symptoms

Characteristic	No. (%) of intervention participants			*P* ^a^
	No SMS returned	1–5 SMS returned	> 5 SMS returned	
Total	105 (49.5)	52 (24.5)	55 (25.9)	-
Sex				
Female	12 (11.4)	13 (25.0)	9 (16.4)	> 0.05
Male	93 (88.6)	39 (75.0)	46 (83.6)	
Age group (years)				
18–40	75 (71.4)	40 (76.9)	45 (81.8)	> 0.05
> 40	30 (28.6)	12 (23.1)	10 (18.2)	
Nationality				
Vanuatu	2 (1.9)	1 (1.9)	5 (9.1)	> 0.05
Other	103 (98.1)	51 (98.1)	50 (90.9)	

^a^ χ^2^ tests were used to compare the response rates of the intervention group by demographic variable.

### Intervention impact

Overall, four people in the intervention group (1.6%) and none in the control group reported symptoms during the quarantine period; the difference was not statistically significant. All four people who reported symptoms received a SARS-CoV-2 test, and none were positive.

### Evaluation of the intervention

The post-intervention evaluation was completed by 92 of the 253 participants in the intervention group (36.3%). Among respondents to the evaluation, 32 (34.8%) were female and 60 (65.2%) were male. The majority of females (27; 84.4%) and males (35; 58.3%) agreed that the instructions were easy to follow (**Table 3**). The majority of females (18; 56.3%) disagreed with the statement that responding by SMS was hard, while the majority of males (32; 53.3%) were neutral about the statement. The majority of females (19; 59.4%) and males (35; 58.3%) agreed that they were comfortable answering the questions.

**Table 3:** Responses to post-intervention evaluation by 92/253 participants in the intervention group

Evaluation category	No. (%) of intervention participants	
	Female (*n* = 32)	Male (*n* = 60)
**Ease and acceptability of the intervention**		
Instructions were easy to follow		
Agree	27 (84.4)	35 (58.3)
Neutral	4 (12.5)	21 (35.0)
Disagree	1 (3.1)	2 (3.3)
Missing	0 (0.0)	2 (3.3)
Responding was hard		
Agree	2 (6.3)	11 (18.3)
Neutral	10 (31.3)	32 (53.3)
Disagree	18 (56.3)	12 (20.0)
Missing	2 (6.3)	5 (8.3)
I felt comfortable answering the questions		
Agree	19 (59.4)	35 (58.3)
Neutral	8 (25.0)	21 (35.0)
Disagree	4 (12.5)	2 (3.3)
Missing	1 (3.1)	2 (3.3)
**Reason for not responding**		
I had technical issues	2 (6.3)	4 (6.7)
I did not receive any messages	1 (3.1)	6 (10.0)
I did not understand the instructions	0 (0.0)	15 (25.0)
I did not activate the SIM card	2 (6.3)	3 (5.0)
I forgot	1 (3.1)	9 (15.0)
Other	8 (25.0)	6 (10.0)
Missing	18 (56.3)	17 (28.3)

Reasons for not responding included not activating the SIM card (females: 2, 6.3%; males: 3, 5.0%), having technical issues (females: 2, 6.3%; males: 4, 6.7%) and other reasons (not further described; females: 8, 25.0%; males: 6, 10.0%).

## Discussion

The detection rate of acute respiratory illness was higher for the intervention group than for the control group. However, it should be noted that the number of people reporting symptoms was low in both groups and is not statistically significant. Globally, there has been a decrease in respiratory specimens testing positive for influenza at sentinel surveillance sites. (*10*) This has also been reported in Vanuatu. (*11*) It is difficult to determine whether the overall low rate of symptom detection reflects underreporting in both the control and intervention groups. In contrast, in 2021 a new model for quarantine monitoring was adopted by the Vanuatu MoH whereby people in quarantine are telephoned to enquire about symptom development at two points during quarantine. From 1 January to 31 May 2021, 1226 people completed quarantine, among whom 5 (0.40%) reported influenza-like illness. This suggests that the SMS intervention may achieve a higher rate of reporting; however, further research is required.

There were several operational issues related to the use of SMS texts in Vanuatu, including challenges to ensuring that the SMS texts sent were received by participants. Reasons why texts were not received include people not activating the SIM cards and the use of an overseas-based bulk SMS service to send the SMS texts. During the post-intervention survey, people who did not respond to the SMS texts indicated that they forgot, had technical issues or did not understand the instructions. Translating the SMS text to Bislama may increase response rates, and the remaining barriers could all potentially be addressed through improved troubleshooting of communication and technology issues. It is unclear whether literacy rates contributed to successful reporting.

Overall, most participants reported that the intervention instructions were easy to follow and they were comfortable responding by SMS. However, a higher proportion of males than females found reporting by SMS to be difficult. This conflicts with the finding that the majority of females and males found the instructions easy to follow, so the challenges in responding by SMS may be due to technical or logistical issues associated with responding by SMS rather than understanding the instructions.

Our results suggest that both SMS and phone monitoring are acceptable methods, but that sufficient systems to identify and troubleshoot technological issues are required. In addition, our findings suggest that the use of one-way SMS rather than two-way (that is, communicating information by SMS without requiring a response) may be successful.

There are some additional limitations to consider. First, the sample size of 540 participants could not be reached and, as a result, the study has reduced statistical power to compare the control and intervention groups. However, we note that the broader objectives of the study were to evaluate the acceptability, efficiency and utility of SMS-based health monitoring, and the study was able to meet this objective. Second, it was not possible to validate whether travellers in the intervention group were truly asymptomatic as opposed to those in the control group; however, participants in the intervention group each had a specimen tested for SARS-CoV-2, and all results were negative. Third, the response rate to the evaluation among participants was low (31%) and, therefore, may not reflect the attitudes of all participants. However, some important findings about ways to improve response rates by SMS in the future were identified.

## Conclusions

The findings of this research highlight that SMS is a useful tool to monitor symptom development among people in quarantine and that the intervention was both easy to understand and acceptable. The intervention achieved a 50% response rate, despite some technical difficulties. The findings show that SMS is not inferior to in-person symptom detection and may be more effective than other methods, such as telephone calls. These findings have implications for COVID-19 responses in resource-limited settings as well as relevance to broader public health programmes that require follow up.
